# Using Simulation and Budget Models to Scale-Up Nitrogen Leaching from Field to Region in Canada

**DOI:** 10.1100/tsw.2001.355

**Published:** 2001-12-11

**Authors:** E.C. Huffman, J.Y. Yang, S. Gameda, R. de Jong

**Affiliations:** Eastern Cereal and Oilseed Research Centre, Agriculture and Agri-Food Canada, Ottawa, ON, Canada

**Keywords:** nitrogen, indicator, water contamination, simulation model, model evaluation

## Abstract

Efforts are underway at Agriculture and Agri-Food Canada (AAFC) to develop an integrated, nationally applicable, socioeconomic/biophysical modeling capability in order to predict the environmental impacts of policy and program scenarios. This paper outlines our Decision Support System (DSS), which integrates the IROWCN (Indicator of the Risk of Water Contamination by Nitrogen) index with the agricultural policy model CRAM (Canadian Regional Agricultural Model) and presents an outline of our methodology to provide independent assessments of the IROWCN results through the use of nitrogen (N) simulation models in select, data-rich areas. Three field-level models — DSSAT, N_ABLE, and EPIC — were evaluated using local measured data. The results show that all three dynamic models can be used to simulate biomass, grain yield, and soil N dynamics at the field level; but the accuracy of the models differ, suggesting that models need to be calibrated using local measured data before they are used in Canada. Further simulation of IROWCN in a maize field using N_ABLE showed that soil-mineral N levels are highly affected by the amount of fertilizer N applied and the time of year, meaning that fertilizer and manure N applications and weather data are crucial for improving IROWCN. Methods of scaling-up simulated IROWCN from field-level to soil-landscape polygons and CRAM regions are discussed.

## INTRODUCTION

Nitrogen (N) has a decisive influence on crop production and environmental contamination. From the point of view of food production, high levels of organic and/or inorganic N fertilizer application rates are required to obtain optimal crop yields. On the other hand, from an environmental point of view, contamination of water by surplus N from agricultural land has gradually increased and has become of concern in many countries[1,2,3]. Therefore, the prediction of the possible impacts of ongoing and changing intensive farming systems on water contamination is a major concern for policy makers, agronomists, and soil scientists. In Canada, an Indicator of Risk of Water Contamination by Nitrogen (IROWCN) was used to estimate the level of risk of soil-mineral N leaching to the groundwater at a regional scale[4,5]. In contrast to the indicator approach, many process-based simulation models are being used to assess the likelihood of N leaching and runoff at both field and agroecosystem levels[6,7]. Integration of either a model or an index into a Decision Support System (DSS) is necessary to carry out broad-area assessments, and this will involve database design, interface design, model integration, and graphic automation. However, an important step prior to integration is the evaluation of output with locally measured data. In this paper, we present the results of our evaluation of three crop simulation models with Canadian field data and present a method to validate the IROWCN index using a simulation model.

## OUTLINE OF THE PROJECT

We are developing a DSS by integrating a general N-budget index (IROWCN) with the Canadian Regional Agriculture Model (CRAM). CRAM is used as an analytical tool by the federal agricultural policy sector in Canada to test the effects of policy scenarios on crop and livestock distributions, and it provides cost- and revenue-optimization estimations of agricultural land and levels of livestock[8]. A Land Use Allocation Model (LUAM) is being developed to allocate CRAM scenario outputs for relatively large areas to smaller soil-landscape units based on land suitability, soil capability, and cropping history. The CRAM scenario outputs, allocated in a rational way to soil-landscapes with the LUAM, will be used as input to calculate IROWCN in order to assess the effect of policy changes on the risk of N contamination of surface and groundwater.

Since the IROWCN index was developed as an agrienvironmental "indicator" and calculated on a soil-landscape polygon level (scale 1:1 million) using general data from the Census of Agriculture and monthly climate normals data, we have found it difficult to test IROWCN in a rigorous fashion. Our current approach is to use a crop-N simulation model to test the IROWCN index in several soil-landscape polygons where the detailed and extensive data required for the simulation model exists. After being tested and improved where possible, the index can then be used with more confidence to evaluate policy scenarios in areas with insufficient data to operate the dynamic models.

This approach also allows us to assess the relationship between the reliability or accuracy of our estimates and the costs (with respect to data needs) of the different approaches. This is of particular importance in integrating economic policy and environmental impacts, since policy makers should know that the relatively low-cost application of IROWCN will provide a certain level of reliability, while increasing that accuracy to a certain, higher level may require significant investment in data enhancement. By comparing the two approaches, not only can the reliability of the index be tested, but the sensitivity of IROWCN to different input data can be evaluated in order to improve the index where possible or to target specific types of data for improvement. An outline of the project is shown in Fig. 1.

## IROWCN INDEX

IROWCN calculations were reported by MacDonald and Gleig[4] in 1996 and Harker[5] in 1997. The IROWCN index is the ratio of the amount of residual N to the amount of excess soil water available and represents an average concentration of N in water leaving the root zone. IROWCN calculations are made at the Soil Landscapes of Canada[9] polygon level. For components relating to crop management (area of crops, fertilizer rates, manure rates, etc.) the Census of Agriculture served as the database, while soils data came from the Soil Landscapes of Canada extended database and the Canadian Ecodistrict Climate Normals Database[10] was used to calculate excess water. What follows is a brief discussion of the steps and key equations used in the IROWCN calculation.

Step one calculates the amount of soil-mineral N in each polygon, in kg, in the root zone (0–90 cm) after harvest using the following simplified equation[11]:

$$N_{res} = \sum_{j=1}^{m} (N_{fert} + N_{man} + N_{fix} - N_{crop} - N_{denit})_j \times A_j \qquad (1)$$

where $j$ = crop type, $N_{res}$ = soil-mineral N in the root zone at harvest (kg), $N_{fert}$ = N added in inorganic fertilizers (kg/ha),

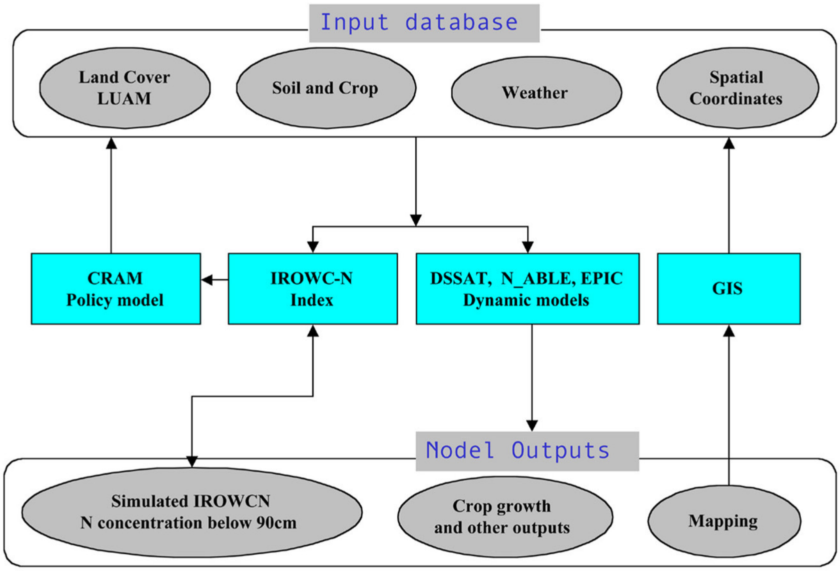

**FIGURE 1**. Flow chart of the CRAM/IROWCN DSS.

$N_{man}$ = N added in manure (kg/ha), $N_{fix}$ = N added by N fixation (kg/ha), $N_{crop}$ = N removed in the harvested crop (kg/ha), $N_{denit}$ = N lost through denitrification (kg/ha and calculated as $0.05 \times N_{man}$), and $A_j$ = area of crop type $j$ in the polygon (ha).

Step two calculates the amount of the residual N lost from the soil profile within each polygon by considering the amount of water available to move it. The water budget includes precipitation (P), potential evapotranspiration (PE), and soil-water holding capacity (AWC). AWC is assumed to be full at planting (April 1), and P is allocated first to fulfill PE needs, with any excess then being added to AWC. Conversely, a deficit between P and PE is drawn from AWC. Water moving out of the root zone occurs only with AWC full and an excess of P over PE. Total N lost is calculated using the equation[11]:

$$N_{lost} = N_{res} \times (P - PE) \div (AWC + (P - PE)) \div A \quad (2)$$

where $N_{lost}$ = N lost from the soil profile (kg/ha), $N_{res}$ = soil-mineral N in the root zone at harvest (kg), $P$ = precipitation (mm), $PE$ = potential evapotranspiration (mm), $AWC$ = Available Water Holding Capacity of the soil[12] (mm), and $A$ = area contributing water in each polygon (ha).

Typically in Canada a moisture deficit occurs through the growing season, and it is not until after harvest that water becomes available to move out of the root zone.

Step three calculates the concentration of N in water leaving the root zone, in mg/l using the equation[11]:

$$\text{IROWCN (mg/l)} = (N_{lost} \times 100) \div (P - PE) \quad (3)$$

Based on the above calculations, IROWCN distribution in 1996 in Canada's humid regions was grouped into three categories (<6, 6 to 14, and >14 mg N/l) and mapped for presentation. An example of the proportion of provincial farmland in each category is presented in Fig. 2.

With the spatial generalizations inherent in IROWCN, it is important that it be tested in a number of locations and assessed for sensitivity under different conditions. Our approach is to compare IROWCN results against a dynamic simulation model in a number of areas where we have sufficient data and spatial detail to employ the model. We are currently evaluating several simulation models for that purpose, and the remainder of this paper outlines our evaluation of crop-growth models, specifically in relation to their N component, using Canadian data.

## DYNAMIC MODELS

Three crop/environmental–growth simulation models — DSSAT, N_ABLE, and EPIC — were selected for comparisons in this study. All these process-based dynamic simulation models include crop growth, water balance, and N dynamic routines; and they all produce daily output for the same state variables as those measured in different field experiments.

DSSAT (Decision Support System for Agrotechnology Transfer) is a family of crop-growth simulation models developed through collaboration between scientists in the U.S. and other countries[14]. It is a process-based dynamic simulation tool that uses many common subroutines (e.g., those for water and N modeling), but different ones for different crop types: CERES for cereals, CROPGRO for grain legumes, and SUBSTOR for root and tuber crops. The current version (3.5) of DSSAT includes more than 16 crops. Particular strong points of the DSSAT models are their plant component and their ability to model different cultivars. During the last 10 years, DSSAT was further developed for rotation applications and integrated with a GIS for spatial applications, e.g., AEGIS and AEGISWIN[15]. Most of the DSSAT crop-growth models have been evaluated successfully in different climatic zones[16,17].

N_ABLE is a crop-N simulation model designed for field vegetable and arable crops in the U.K. It was developed at Horticulture Research International, U.K. to predict the uptake and response of vegetable crops to soil and fertilizer N[18,19]. N_ABLE has been tested extensively against field data in England and western Europe during the last 10 years. It has been shown to give broad agreement for a wide range of crops and conditions[20], although some areas have been identified which require further investigation. More detailed evaluations and modifications of N_ABLE were given by Yang et al.[21]. For the work reported in this paper, soybeans have been integrated into a modified version of N_ABLE, and the results are presented.

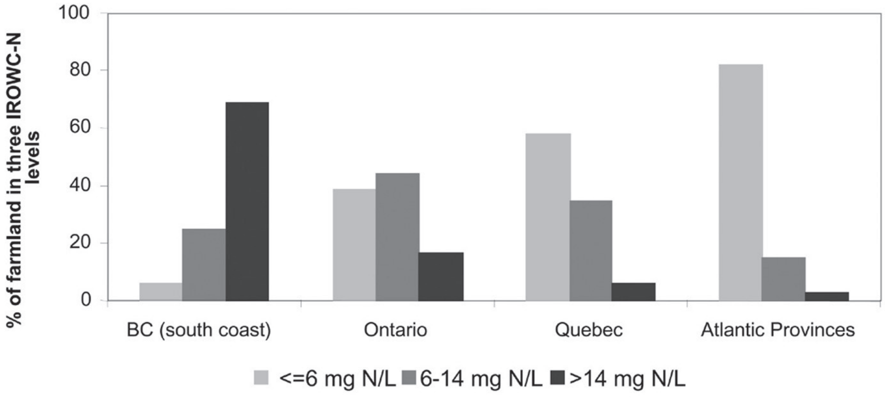

**FIGURE 2.** Indicator of risk of water contamination by N on farmland in Canada's humid regions in 1996[13].

EPIC (Environmental Policy Integrated with Climate, formerly known as Erosion Productivity Impact Calculator) is a soil-erosion simulation model originally developed in the 1980s to predict the relationship between soil erosion and soil productivity throughout the U.S.[22]. This model integrates the major processes that occur in the soil/crop/atmosphere–management system, including hydrology, weather, wind and water erosion, nutrient cycling, plant growth, soil temperature, tillage operations, and economics. EPIC uses a single model for simulating all crops, with each crop (currently more than 20) having its own unique parameter values. The crop-growth model uses radiation-use efficiency in calculating photosynthetic production of biomass. The computed potential biomass is adjusted daily for stress from water, temperature, nutrients (N and P), and soil aeration. Crop yields are estimated by multiplying the aboveground biomass at maturity by a water-stress–adjusted harvest index for a particular crop. The model runs on a daily time-step at the scale of a single field and is well suited to multiyear simulations. Recent applications of the EPIC to simulate soil-N dynamics on a large scale have been carried out by many researchers[23,24,25].

## CASE STUDIES: FIELD SIMULATION

Four years of maize and soybean data were collected from field experiments. The maize experiments were carried out at the Central Experimental Farm in Ottawa from 1993 to 1996[26], while the soybean data are from a variety of trials carried out at Harrington, PEI between 1990 and 1993. The general experimental conditions are listed in Table 1.

### Soybean Simulation

Examples of biomass simulations of soybean at Harrington, PEI using the three models are given in Fig. 3. A linear regression between simulated and measured data was carried out for statistical evaluation.

### Maize Simulation

Maize simulation was performed using only DSSAT (CERES-MAIZE) and N_ABLE. Simulated aboveground biomass and grain yield of maize and soil-mineral N in the 0- to 90-cm layer are compared with data measured at Ottawa in 1993, 1994, 1995, and 1996. Fig. 4 provides a comparison between simulated and measured biomass and soil-mineral N at harvest in each year.

### Results and Discussion

Figure 3 shows that the DSSAT and N_ABLE models give reasonable simulations of aboveground soybean biomass over the growing period, but that EPIC overestimates the biomass during

**TABLE 1**
**General Conditions of Field Experiments in Two Different Locations**

| | Soybean (Harrington, PEI) | | | | Maize (Ottawa) | | | |
|---|---|---|---|---|---|---|---|---|
| | 1990 | 1991 | 1992 | 1993 | 1993 | 1994 | 1995 | 1996 |
| ***Crop Cultivation Data*** | | | | | | | | |
| Cultivars | Baron | | | | Pioneer 3902 | | | |
| Growth period (days) | 122 | 131 | 144 | 144 | 154 | 146 | 154 | 151 |
| Density (plant/ha) | 750,000 | | | | 65,284 | | | |
| N applied (kg/ha) | 40 | | | | 200 | | | |
| ***Soil Condition Before Planting*** | | | | | | | | |
| Soil-mineral N (kg N/ha) | 35 | | | | 52 | 48 | 48 | 55 |
| Soil type | Fine sandy loam | | | | Tile-drained fine loam | | | |
| Soil pH in water | 5.7 | | | | 6.5 | | | |
| Sand:silt:clay content (%) | | | | | 39:27:34 | | | |
| Bulk density (0–30 cm) | 1.50 | | | | 1.25 | 1.25 | 1.32 | 1.38 |
| ***Weather Conditions in the Growth Period*** | | | | | | | | |
| Lat./long. | 46°20′N / 63°10′W | | | | 45°22′N / 75°43′W | | | |
| Solar radiation (MJ/m$^2$/day) | 2368 | 2213 | 2300 | 2372 | 2650 | 2631 | 2778 | 2518 |
| Precipitation (mm) | 388 | 347 | 347 | 355 | 441 | 462 | 532 | 521 |
| Potential evap. (mm) | 440 | 402 | 402 | 423 | 496 | 493 | 451 | 480 |
| Accu. temp. > = 8°C | 2206 | 2045 | 2045 | 2176 | 2497 | 2483 | 2745 | 2555 |

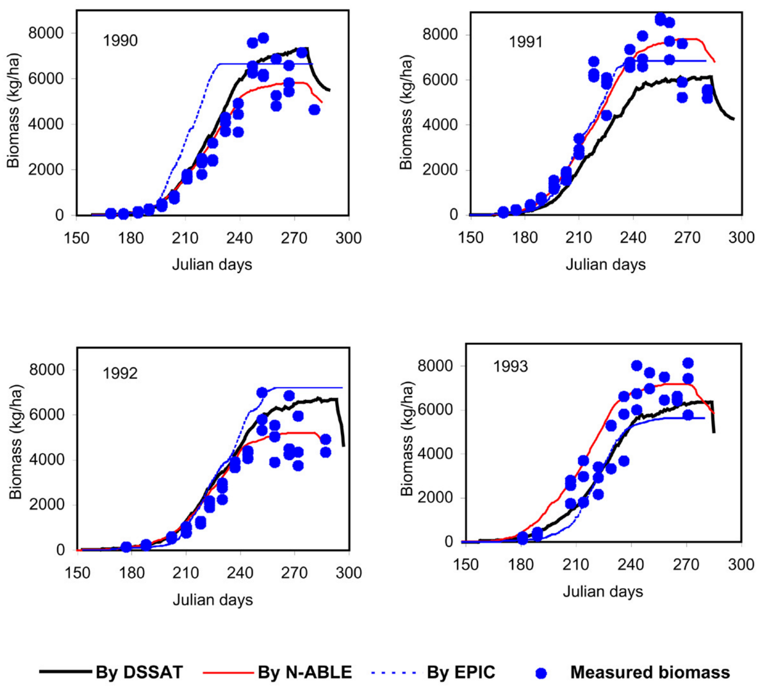

**FIGURE 3**. Comparison of simulated aboveground biomass of soybean using DSSAT, N_ABLE, and EPIC vs. measured data at Harrington, PEI, 1990 to 1993.

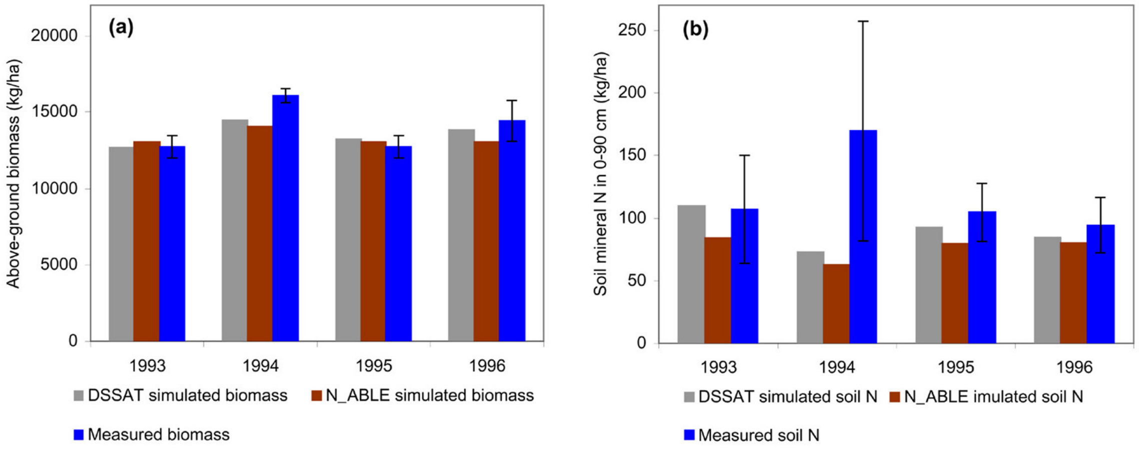

**FIGURE 4.** Comparison of simulated (DSSAT and N_ABLE) and measured (a) aboveground biomass of maize and (b) soil-mineral N in the 0- to 90-cm layer at harvest with 200 kg N/ha, Ottawa, 1993 to 1996. Vertical bars represent the standard deviation of three replicates.

the middle part of the growing season and estimates maximum biomass 4 to 6 weeks earlier than measured. DSSAT and N_ABLE simulated a decline in biomass after physiological maturity due to the fact that they encompass eight and four growth stages, respectively, while EPIC does not account for individual growth stages. A linear regression $y_i = bx_i$, where $y_i$ is the ith measured biomass and $x_i$ is the ith simulated biomass, was used to test the agreement between simulated and measured biomass. Biomass of soybean was measured 47, 48, 41, and 36 times in 1990, 1991, 1992, and 1993, respectively. The regression equation and $R^2$ values for the DSSAT model are listed as follows: y = 0.87x, $R^2$ = 0.94 (1990); y = 1.36x, $R^2$ = 0.74 (1991); y = 0.81x, $R^2$ = 0.89 (1992); and y = 1.23x, $R^2$ = 0.86 (1993). The coefficient b indicates that DSSAT-SOYGRO slightly overestimated biomass in 1990 and 1992 (b < 1) and slightly underestimated biomass in 1991 and 1993 (b > 1). $R^2$ values show the goodness of fit between simulated and measured data. The best fit is in 1990, and the poorest fit is in 1991. A regression equation using all 4 years of data ranges from y = 1.05x with $R^2$ = 0.73 (DSSAT) to y = 1.01x with $R^2$ = 0.85 (N_ABLE) and y = 0.94x with $R^2$ = 0.71 (EPIC). The results show that although DSSAT and EPIC also give reasonable coefficients, the best fit in this situation is provided by N_ABLE.

The results of the maize simulations indicate that both DSSAT and N_ABLE simulate maize biomass and yield accurately throughout the growing period and also give reasonably accurate soil-mineral N simulations for a fertilization rate of 200 kg N/ha. The difference between simulated and measured soil N was < ±1 sd. Fig. 4 shows that all differences between simulated and measured biomass and soil-mineral N at harvest are less than the experimental errors except for soil-mineral N in 1994. The reason for the discrepancy in 1994 is that (1) the models generally underestimate soil-mineral N in the latter part of the growing period and (2) the measured data suffer from experimental error as evidenced by the larger error bar for 1994 in Figure 4(b).

From the above analysis we conclude that any of the three models — DSSAT, N_ABLE, and EPIC — can be used to simulate crop growth and soil N dynamics with reasonable accuracy in Canada, although detailed evaluation of the three models is needed for other crops, soils, and climates.

## SIMULATION OF IROWCN OUTPUT USING N_ABLE

Based on logistical considerations and the work performed so far, the model of choice for validating IROWCN in our DSS project is N_ABLE. This is essentially due to several factors: (1) the need for only daily mean temperature, rainfall, and potential evaporation (no need for solar radiation, which is not available from some weather stations in Canada); (2) the fact that N_ABLE is a more simplified model that seems to perform satisfactorily in modeling detailed N and water-movement dynamics, yield, and biomass; and (3) our resident expertise with that model and the need to integrate and modify the model extensively for use in the DSS. An example of the use of N_ABLE to simulate IROWCN at the field level and the approach to be used in selected, data-rich soil-landscape and CRAM region levels is presented below.

### IROWCN at the Field Level

One of the outputs of N_ABLE is soil-mineral N leached below the 90-cm soil layer ($N_{leach}$) in kg/ha. We can use this value to represent the soil N loss from the leaching process by assuming that $NO_3$–N leached below the 90-cm layer is unlikely to be taken up by crops. We can also obtain estimates of the amount of soil water leached below the 90-cm layer. Based on Eq. 3 above, IROWCN can be simulated with N_ABLE using measured input parameters on a daily step as follows:

$$IROWCN_s(t) = N_{leach}(t) \times 100 / W_{leach}(t) \quad (4)$$

where $IROWCN_s$ = simulated IROWCN using N_ABLE (mg/l), $N_{leach}$ = accumulated soil N leached below the 90-cm soil layer (kg/ha), $W_{leach}$ = accumulated soil water leached below the 90-cm soil layer (mm), and $t$ = day (1 to 365).

Results of the N_ABLE simulations of IROWCN using 1993 data from the maize field at Ottawa show that at a fertilization rate of 300 kg N/ha, soil-mineral N below the 90-cm soil layer remains relatively low and constant over the growth period and then rises after harvest (data not shown). This is consistent with the assumptions used in calculating IROWCN.

IROWCN simulations using 4 years of data and two fertilization rates from the maize experiments at Ottawa as input to N_ABLE show that two levels of N fertilizer result in very different simulated IROWCN levels after harvest (Fig. 5). For example, with 150 kg N/ha as shown in 5(a), simulated IROWCN declines below 10 mg/l after harvest, probably due to more rainfall later in autumn, while, with 300 kg N/ha as shown in 5(b), simulated IROWCN increases dramatically after harvest due to a higher amount of residual N in the soil. As expected, a higher N fertilization rate results in greater potential of water contamination. The implications of this comparison are that data pertaining to fertilization rates, and especially variability in rates across a landscape, are important in the application of IROWCN.

### IROWCN at the Soil-Landscape Level

The Census of Agriculture database for Ontario shows 14 crop types in a total of 381 soil-landscape polygons. Using Eq. 4 and the census database, IROWCN at the soil polygon level can be simulated as follows:

$$IROWCN_s(i) = \sum_{j=1}^{14} IROWCN_s(i, j) \times A(i, j) \div \sum_{j=1}^{14} A(i, j) \quad (5)$$

where $i$ = soil polygons (1 to 381), $j$ = crops in each soil polygon (1 to 14), $IROWCN_s(i, j)$ = simulated soil N concentration below the 90-cm layer in the $i$th soil polygon under the $j$th crop (kg/ha) (calculated using Eq. 4), and $A(i, j)$ = the $j$th crop area (ha) in the $i$th soil polygon.

### IROWCN at the CRAM Region Level

For application of the socioeconomic model CRAM, Ontario farmland is divided into 10 regions. IROWCN in CRAM regions can be simulated based on the results for soil polygons (Eq. 5) as follows:

$$IROWCN_s(c) = \sum_{i=1}^{c_i} IROWCN_s(i) \times A(i) / \sum_{i=1}^{c_i} A(i) \quad (6)$$

where $c$ = CRAM regions (1 to 10), $c_i$ = the number of soil polygons in each CRAM region, $IROWCN_s(i)$ = simulated soil N concentration below the 90-cm layer in the $i$th soil polygon (mg/l) (calculated using Eq. 5), and $A(i)$ = crop areas in the $i$th soil polygon.

At this point in time, the DSS project is still a work in progress, but work so far indicates that the simulation model N_ABLE is appropriate for application in Canada and that it is suitable for our work on scaling-up field data to regional levels in order to test and improve IROWCN. It is also apparent that the IROWCN index can be improved or replaced through detailed work with N_ABLE, thus improving our confidence in the results. For example, national application of IROWCN will allow for the identification of “hot spots”, which can then be the focus of improved data (especially weather) collection to allow for more temporally precise outputs (i.e., monthly or weekly). Similarly, other data sources can be used to identify regions of intensive livestock or vegetable production where the regional nature of IROWCN masks localized issues that would be better

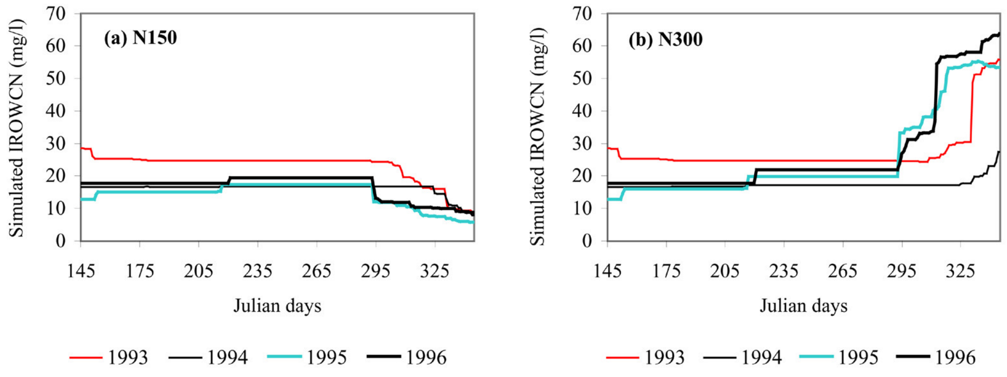

**FIGURE 5.** Change of simulated IROWCN over time in a maize field receiving (a) 150 kg N/ha and (b) 300 kg N/ha in 1993, 1994, 1995, and 1996 at Ottawa.

analysed with N_ABLE simulations. Application of the revised index over a large area, and progress with its integration with CRAM, will be reported in future papers.

## ACKNOWLEDGEMENTS

The authors wish to thank A. Bootsma and B.L. Ma of the Eastern Cereal and Oilseed Research Centre, Agriculture and Agri-Food Canada, Ottawa for providing field data for soybeans and maize, respectively.

| | | |
|---|---|---|
| Received: | July | 17, 2001 |
| Revised: | October | 26, 2001 |
| Accepted: | November | 2, 2001 |
| Published: | December | 11, 2001 |
